# Machine learning-based prediction of polyvinyl alcohol product viscosity and design of optimal process conditions

**DOI:** 10.1007/s44211-026-00939-5

**Published:** 2026-06-15

**Authors:** Ryota Nomura, Yoshihito Yamauchi, Hiroto Misawa, Kosuke Nishigaya, Satoshi Ooyama, Hiromasa Kaneko

**Affiliations:** 1 Department of Applied Chemistry, School of Science and Technology, Meiji University, 1-1-1 Higashi-Mita, Tama-ku, Kawasaki, Kanagawa 214-8571 Japan; 2 Mitsubishi Chemical Corporation, 3-10 Ushiodori, Kurashiki, Okayama 712-8054 Japan

**Keywords:** Machine learning, Soft sensor, Polyvinyl alcohol, Viscosity, Prediction, Design process conditions

## Abstract

**Abstract:**

We developed a soft sensor to predict polyvinyl alcohol viscosity and designed process conditions to reach the target range. Using squared and cross terms and time-series data improved prediction accuracy. In the case study, optimal conditions brought off-target products into range.

**Graphical abstract:**

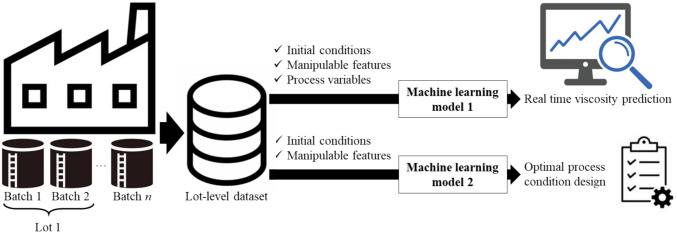

## Introduction

Polyvinyl alcohol (PVA) is used in various applications such as adhesives, binders, and films because of its low cost, non-toxicity, and ease of use [1]. In some PVA production plants, polymerization is performed in batch operations and then the polymerization products from several batches are mixed to form a single PVA product lot. To be shipped as a product, the PVA must meet the specified degree of polymerization for each grade. Therefore, the plant must be operated under conditions that ensure the degree of polymerization is maintained within the specified range. Product viscosity, which is correlated with the degree of polymerization, can be used as an operation index. In such cases, a target range is set inside the specified range of product viscosity to ensure operation within the specifications.

Although it is desirable to maintain product viscosity within the target range during operation, viscosity is prone to variation because the degree of polymerization is easily affected by process conditions, and in some cases the viscosity of the final product may fall outside the desired range. In addition, there is generally a time lag between polymerization and measurement of product viscosity because of the multiple processes involved. It is essential to estimate product viscosity before formation to achieve efficient control of viscosity. One effective approach is to use a soft sensor, which is a regression model in which a difficult-to-measure variable y is estimated from an easy-to-measure variable x by constructing a mathematical model y = f(x). As newly measured values of x are input into the model, the value of y can be predicted in real time.

Two primary approaches are commonly used in the development of soft sensors: theoretical modeling based on physical equations [2] and data-driven modeling using machine learning techniques. In the latter approach, soft sensors are developed using historical process data. Recently, machine learning-based soft sensors have gained widespread acceptance and been successfully applied to various industrial processes, such as real-time monitoring of polyethylene production [3] and quality prediction of cell culture processes [4]. Considerable progress has been made in the development of high-performance soft sensors, including models capable of accurate prediction even when faced with missing data [5] or limited sample size [6, 7]. Models capable of predicting multiple quality attributes simultaneously have also been developed [8]. In cases where available data are limited, the appropriate amount of real or pseudo-sample data required to ensure reliable model construction has been investigated [9].

Optimization of soft sensor design is currently an active research subject. Various optimization methods have been proposed to improve prediction accuracy [10]. In addition, soft sensors that account for process dynamics have been explored. For example, phase-wise modeling—where the process is divided into several phases and a separate model is constructed for each phase—has been shown to improve the prediction performance of complex processes [11, 12]. This approach has also been shown to be effective for inherently multiphase processes, such as the multilayer batch process, where phase-specific modeling has resulted in improved prediction accuracy [13]. In one such study on multi-layer batch processes, a highly interpretable model with high predictive performance was developed by introducing a normalization term during model construction, allowing the elimination of irrelevant variables at each stage [14]. In addition, research effort has focused on predicting future process behavior during the operation of individual batches and estimating final product quality using intermediate data [15, 16]. These advances facilitate real-time quality control and enable predictive decision making at different batch times.

The constructed models can be used for predicting product quality, investigating the influence of process variables on quality, process control, and quality management. For example, predictive models have been developed for polymerization processes to analyze the effect of input variables on product quality [17, 18]. Such models have also been applied to process control in a distillation column [19] and batch processes [20–22]. One approach to achieve quality control is the inverse analysis of soft sensors. The constructed soft sensor model is used to identify the set of input and operating conditions that are expected to yield the desired product quality. Optimal input conditions can be derived through this inverse analysis, thereby facilitating process optimization [23]. Several studies have applied inverse analysis to batch processes, enabling the identification of optimal operating variables and design of time-series input profiles [24, 25].

The objectives of this study are to develop a soft sensor that predicts product viscosity at the end of polymerization and to determine optimal process conditions to achieve the target product viscosity. Achieving these objectives will eliminate the time loss from polymerization to viscosity measurement and maintain the product viscosity within the target range. The soft sensor is developed by constructing a model that predicts product viscosity based on information integrated from the multiple batches in a single lot. In the target plant, many grades of products are provided through multiple batch operations and the number of batches per lot varies. Because product viscosity is measured on a per-lot basis, one viscosity value corresponds to multiple batch runs. Therefore, the model is designed to predict the viscosity of a lot by aggregating information from all its constituent batches. This enables the estimation of product viscosity immediately after the polymerization process for the lot is completed. In addition to feedstock data and process variables, time-series data from each batch operation are incorporated, resulting in a soft sensor with high predictive accuracy.

For the design of manufacturing conditions, the optimal manipulable variables are determined through inverse analysis of the soft sensor. In this study, the final batch in the series of batch operations that constitutes one product lot is designed so that the overall product viscosity of the lot will meet the target value. This approach enables control of product lots whose viscosity is predicted to deviate from the target range by adjusting the final batch to bring the lot within specification. The effectiveness of the proposed method is verified by designing process conditions that modify the viscosity of the final batch to ensure that the viscosity of the lot is within the target range.

## Methods

**Fig. 1:**
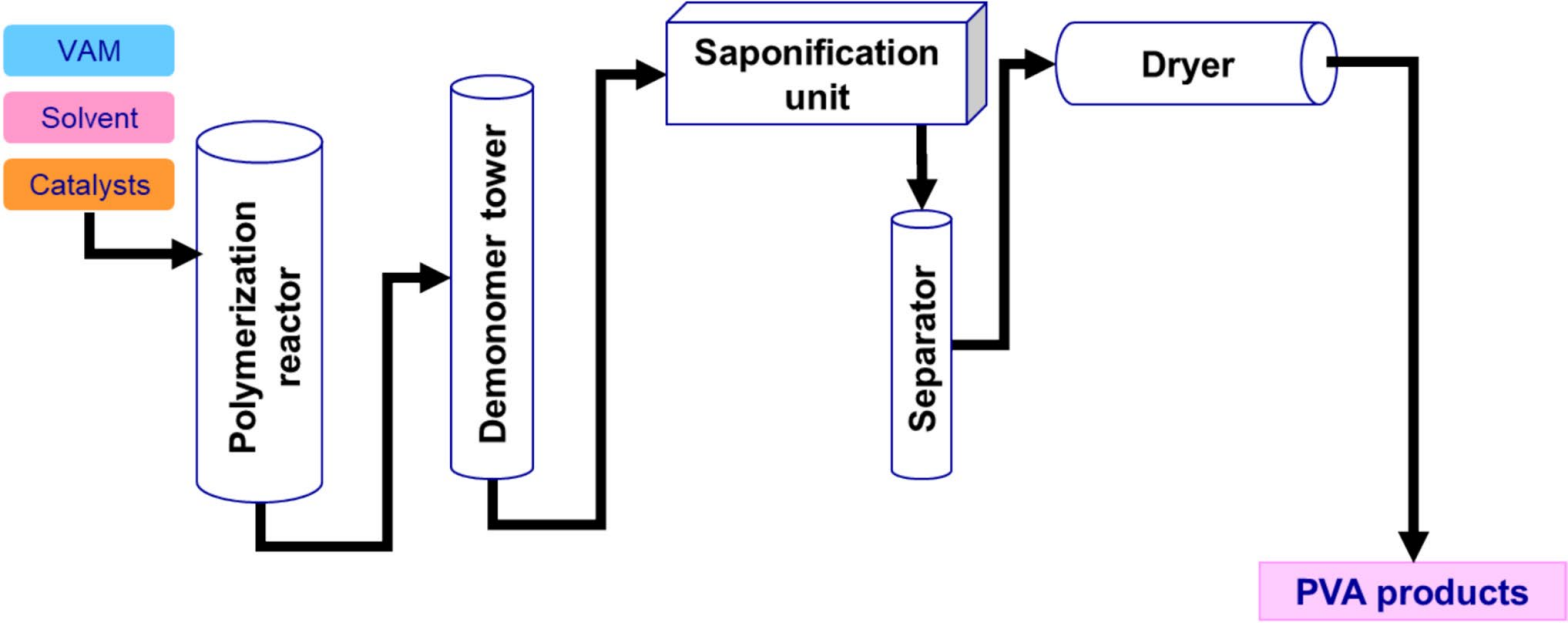
Process flow diagram of a PVA production plant

In this study, operational data from the PVA production plant of Mitsubishi Chemical Corporation were used. Figure 1 shows the process flow diagram of the PVA production plant. The raw material, vinyl acetate monomer (VAM), was polymerized in a polymerization tank, followed by saponification in a saponification unit to produce PVA. The product viscosity was measured when the PVA product was obtained.

### Dataset

The dataset consisted of 824 batch operation records corresponding to 140 product lots. The dataset contained information on feedstocks, impurities, catalysts, and process variables. The process variables included both endpoint values measured at the end of polymerization and time-series data collected continuously during the batch operation. Among the endpoint parameters, final agitation power served as a target variable, and the polymerization process was terminated when the final agitation power reached its predefined value. The dataset also included variables that defined the initial conditions prior to polymerization, as well as both controllable and uncontrollable variables. The time-series data were measured continuously and thus included values before, after, and during the polymerization reaction. However, only the data collected during the reaction were used.

### Feature extraction from time-series data

**Fig. 2:**
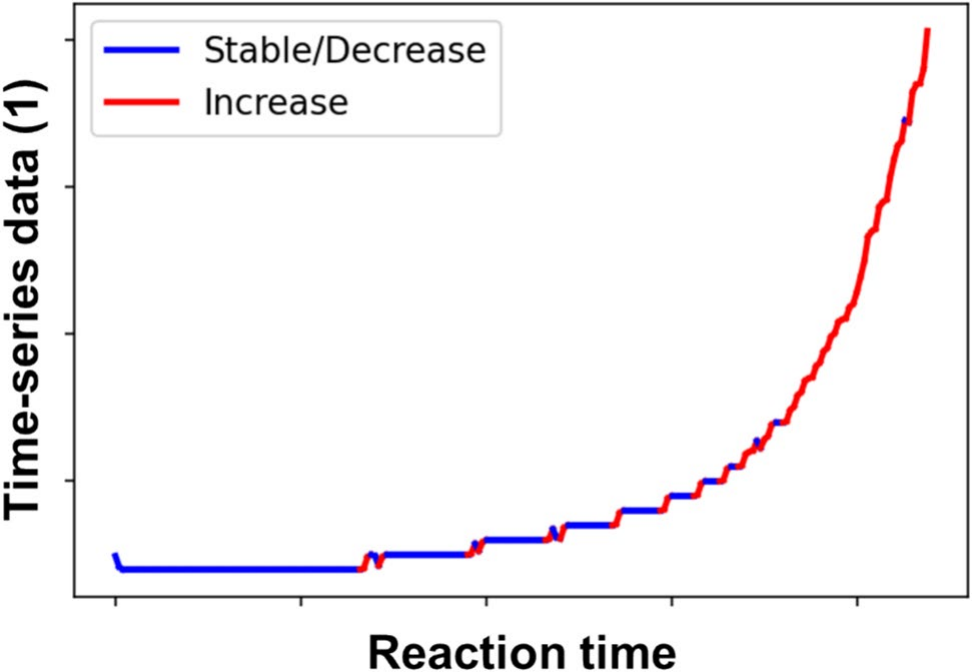
Trends in time-series data (1). The red lines show when time-series data (1) increase

Variables other than time-series data yielded a single value per batch run, whereas time-series data consisted of continuously measured values, resulting in multiple data points per batch. Moreover, because the duration of each batch operation varied, the number of time-series data points also differed from batch to batch. To handle this variability, we used the tsfresh python package [26] to extract features from the time-series data. Using tsfresh, a fixed number of statistical and mathematical features is automatically generated from time-series data, including basic statistics, such as mean and median, as well as more advanced metrics including the number of data points exceeding the mean. In addition, for each time-series variable, cumulative values are computed. For the time-series data (1), which are considered to have a strong influence on the polymerization reaction, both the total and average of the positive changes were calculated and used as features. When computing changes, only the values representing positive changes were extracted—because these are considered important—whereas negative changes were ignored. Figure 2 shows the time-series data (1) for a single batch operation. In this figure, only the positive changes, highlighted in red, were extracted, and their total and average were calculated.

To improve the accuracy of the prediction model, squared terms and cross terms were added for each variable other than time-series data. A summary of the variables and the extracted features used to construct the soft sensor models is provided in Table 1.

**Table 1:** Variables and extracted features used for soft sensor construction

Category	Variable	Unit	Number of features
Feedstocks	Mixture of VAM and MeOH	kg	1 feature
	Ratio of newly supplied VAM	(%)	1 feature
	Feed weight ratio of MeOH/VAM	–	1 feature
Impurities	Impurity (1)	ppm	1 feature
	Impurity (2)	ppm	1 feature
	Impurity (3)	ppm	1 feature
	Impurity (4)	ppm	1 feature
	Impurity (5)	ppm	1 feature
	Impurity (6)	ppm	1 feature
Catalysts	Initial catalyst loading	kg	1 feature
	Total catalyst loading	kg	1 feature
Process variables endpoint values	Atmospheric pressure	mmHg	1 feature
	Reaction time	min	1 feature
	Final agitation power	kW	1 feature
	Polymerization reactor temperature	°C	1 feature
	Paste tank temperature	°C	1 feature
	Residence time	min	1 feature
	Resin content reading	(%)	1 feature
	Resin content	(%)	1 feature
Squared term			19 features
Cross term			171 features
Process variables Time-series data	Time-series data (1)	kW	783 features
	Time-series data (2)	rpm	783 features
	Time-series data (3)	(%)	783 features
	Time-series data (4)	kPaG	783 features
	Time-series data (5)	kPaG	783 features
	Time-series data (6)	kPaG	783 features
	Time-series data (7)	°C	783 features
	Time-series data (8)	°C	783 features
	Time-series data (9)	°C	783 features
	Time-series data (10)	°C	783 features
	Time-series data (11)	°C	783 features
	Cumulative values of each time-series variable		11 features
	Change in agitation power	kW	2 features

### Integration of information from multiple batches

The target plant produced single lots of products through multiple batch operations, and the product viscosity is determined for the integrated lot as a whole, rather than being measured for each individual batch. Therefore, it was necessary to integrate the information from multiple batches that made up a single lot and associate the integrated information with the corresponding product viscosity. To achieve this, we collected all batches belonging to the same lot and calculated the average, maximum, minimum, and median values for each parameter. These integrated statistics were then used as variables for constructing the soft sensors.

### Feature selection

Variables that had the same value for more than 90% of the samples were removed because they held little informational value. Subsequently, the feature selection method Boruta [27] was used to identify variables that were important for predicting product viscosity.

### Handling of missing values

Missing values for one of the endpoint process variables meant that some of the variables selected by the Boruta algorithm also had missing values. To address this, we applied six different imputation methods, as summarized in Table 2, and evaluated prediction accuracy by constructing soft sensors for each method.

**Table 2:** Strategies used to handle missing values

How to handle missing values	Details
Feature removal	Remove features with missing values
Sample removal	Remove samples with missing values
Zero-value imputation	Impute missing values with zero
Mean-value imputation	Impute missing values with the mean
Mode-value imputation	Impute missing values with the mode
iGMR-based imputation	Impute missing values using predictions from iGMR

### Soft sensor modeling

The selected variables were used to construct a regression model y = f(x) using machine learning, where the variables with imputed missing values served as explanatory variables x, and the product viscosity was the target variable y. Double cross-validation (DCV) was used to repeatedly split the data into training and test sets, and the predictive performance was evaluated using various regression methods to identify the optimal approach. The following regression methods were considered: partial least squares (PLS), ridge regression (RR), least absolute shrinkage and selection operator (LASSO), elastic net (EN), linear support vector regression (LSVR), non-linear support vector regression (NLSVR), decision tree (DT), random forest (RF), extreme gradient boosting (XGBoost), gradient boosting decision tree (GBDT), and Gaussian process regression (GPR). Prediction performance was evaluated using the metrics of coefficient of determination (R^2^), mean absolute error (MAE), mean absolute percentage error (MAPE), and root mean squared error (RMSE).

### Design of process conditions

Although inverse analysis of soft sensors can be used for the design of process conditions, the soft sensors constructed above are not suitable for this purpose because their explanatory variables include uncontrollable factors. Therefore, a soft sensor for process condition design was developed using only the initial condition variables determined prior to polymerization and other controllable variables as explanatory variables. The initial and controllable variables used to develop this soft sensor are listed in Table 3.

**Table 3:** Initial condition variables and controllable variables

Category	Variable	Unit
Initial condition variables	Mixture of VAM and MeOH	kg
	Ratio of newly supplied VAM	(%)
	Atmospheric pressure	mmHg
	Impurity (1)	ppm
	Impurity (2)	ppm
	Impurity (3)	ppm
	Impurity (4)	ppm
	Impurity (5)	ppm
	Impurity (6)	ppm
Controllable variables	Initial catalyst loading	kg
	Total catalyst loading	kg
	Feed weight ratio of MeOH/VAM	–
	Final agitation power	kW

To improve the accuracy of the prediction model, squared terms and cross terms were added for each variable. As for the soft sensor described above, it was necessary to integrate information from the multiple batches that constituted a single lot. Therefore, for each lot, multiple batches were collected and the mean, maximum, minimum, and median values of all features were calculated and used as variables. Next, variables with the same value for more than 90% of the samples were removed and variable selection was performed using Boruta. A regression model y = f(x) was then constructed using machine learning, where the selected variables served as explanatory variables x and product viscosity was the target variable y. As in the previous case, predictive performance was evaluated using various regression methods through DCV, and the optimal method was selected. The evaluation metrics used were R^2^, MAE, MAPE, and RMSE. The soft sensor constructed here used only the initial condition variables and controllable variables, making it suitable to predict product viscosity prior to polymerization. This model was applied to process condition design.

In the design of process conditions, the final batch of operations constituting a single lot was designed. For the design method, given a batch with fixed initial condition values, various values were assigned to the controllable variables within those initial conditions to generate virtual samples. Then, the same explanatory variables x used to construct the soft sensor for process condition design were extracted from these samples and input into the soft sensor to calculate the predicted viscosity for all virtual samples. The virtual sample with a predicted viscosity meeting the target value was selected, and the values of the controllable variables for that sample were designated as the optimal process conditions.

## Results and discussion

### Predicting product viscosity

The variables selected using the Boruta algorithm included the ratio of newly supplied VAM, ester-type impurity (3), conjugated-type impurity (6), and paste tank temperature among the original variables. Among the squared and cross-term features, many were associated with the mixture of VAM and MeOH, the ratio of newly supplied VAM, aldehyde-type impurity (1), ester-type impurity (3), and conjugated-type impurity (6), indicating their strong contribution to the model. In addition, all time-series data were selected. These parameters are considered to influence product viscosity.

Polymer solution viscosity is strongly affected by polymer concentration, temperature, and solvent type [28]. The selection of feedstock-related variables, impurity-related variables, and paste tank temperature is consistent with prior knowledge, as these factors are known to influence viscosity, indicating that the results are reasonable. Although the feed weight ratio of MeOH/VAM is also expected to affect viscosity, it was not selected in this study. This is likely because the dataset used in this study consisted of a single grade of PVA, resulting in limited variation in the feed weight ratio of MeOH/VAM. Consequently, it was not identified as an important variable by the Boruta algorithm. The selection of a large amount of time-series data suggests that time-series information strongly affects product viscosity. It is also noteworthy that Boruta did not select any controllable variables as a result.

To evaluate the effectiveness of time-series data and squared and cross terms, the following four types of datasets were used. (1) Dataset without time-series data, squared terms, and cross terms, (2) Dataset with time-series data but without squared terms and cross terms, (3) Dataset without time-series data but with squared terms and cross terms, and (4) Dataset with time-series data, squared terms, and cross terms.

**Fig. 3:**
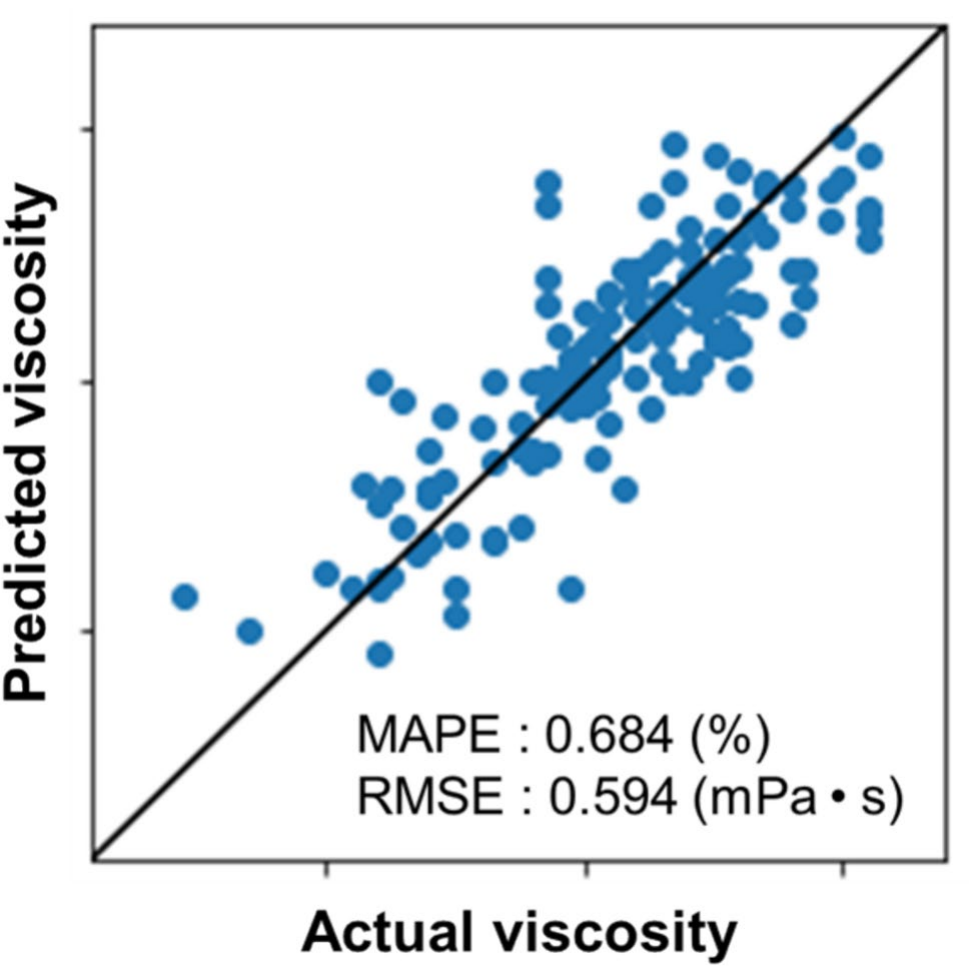
Plot of actual versus predicted values of product viscosity by a soft sensor using a dataset without time-series data, squared terms, and cross terms

**Fig. 4:**
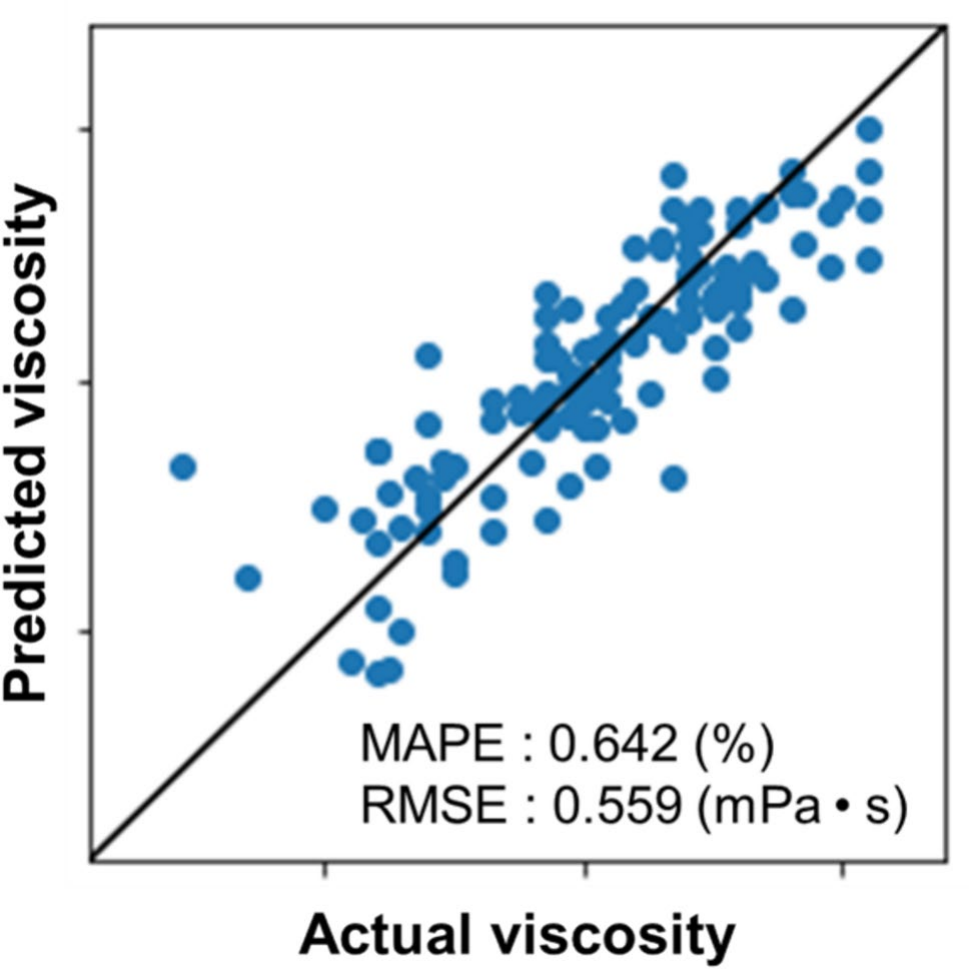
Plot of actual versus predicted values of product viscosity by a soft sensor using a dataset with time-series data and without squared terms and cross terms

**Fig. 5:**
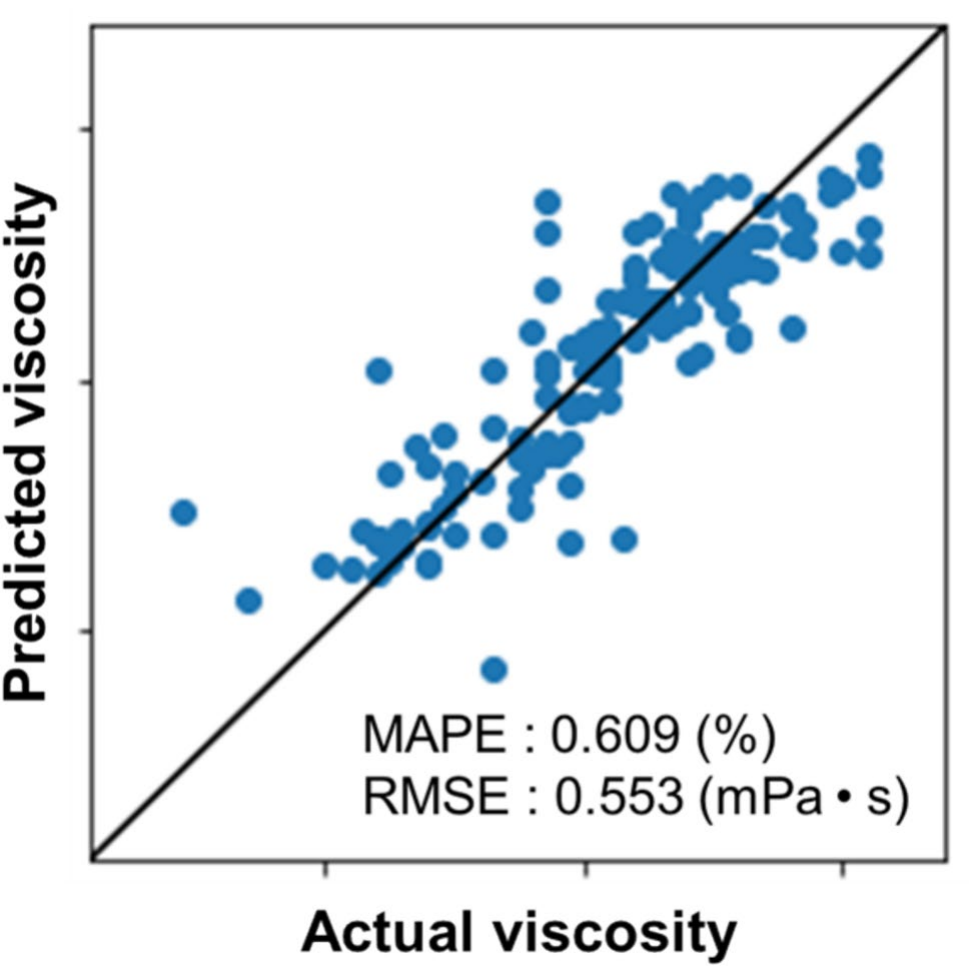
Plot of actual versus predicted values of product viscosity by a soft sensor using a dataset without time-series data and with squared terms and cross terms

**Fig. 6:**
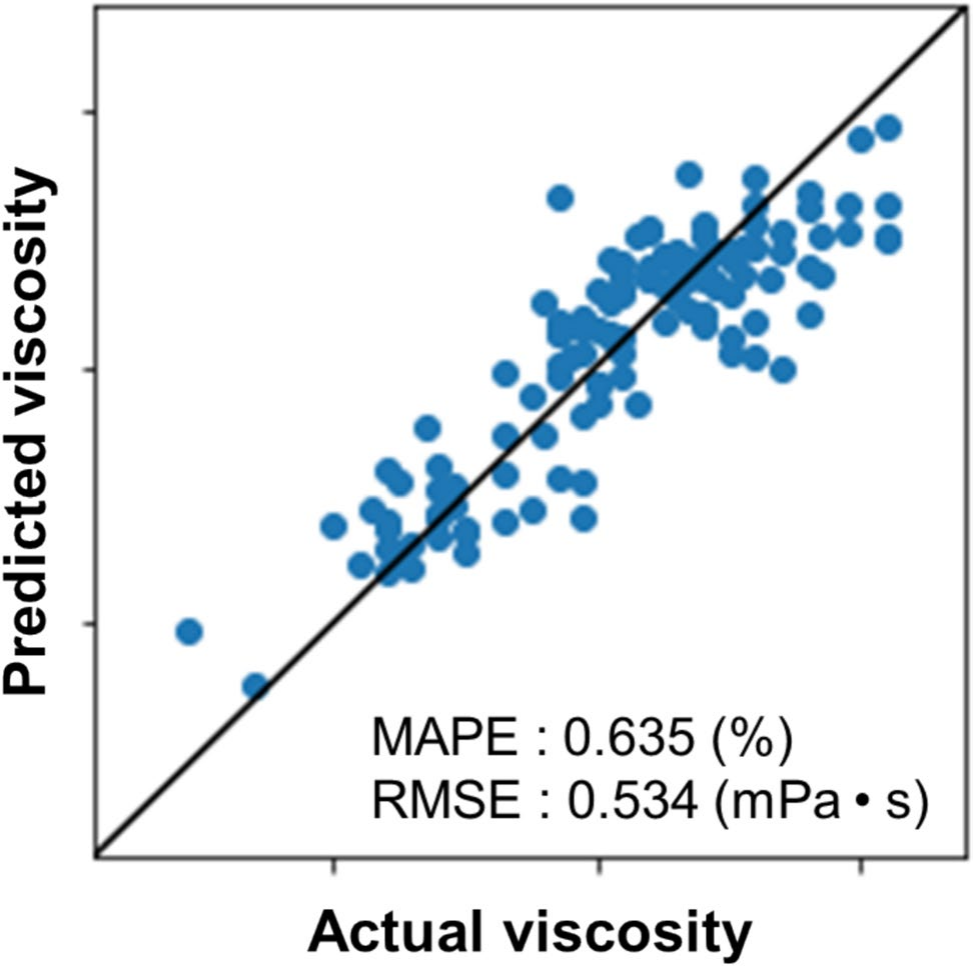
Plot of actual versus predicted values of product viscosity by a soft sensor using a dataset with time-series data, squared terms, and cross terms

For each dataset, missing values were handled using the six methods listed in Table 2. Soft sensors were constructed using the 11 regression analysis methods described in Sect. "Soft sensor modeling". The resulting prediction accuracies are summarized in Table 4. For the soft sensor with the highest prediction accuracy in each dataset, plots of actual values versus predicted values are shown in Fig. 3–6. As presented in Table 4, the XGBoost model built using the dataset with time-series data, squared terms, and cross terms (samples containing missing values were removed) achieved the highest prediction accuracy.

**Table 4:** Comparison of prediction accuracy for 24 combinations of four datasets and six methods used to handle missing values

Dataset	Handling missing values	Method	R^2^	MAE (mPa•s)	MAPE (%)	RMSE (mPa•s)
Dataset without time-series data, squared terms and cross terms	Feature removal	NLSVR	0.687	0.452	0.684	0.594
	Sample removal	RF	0.618	0.517	0.783	0.668
	Zero-value imputation	GBDT	0.633	0.495	0.749	0.643
	Mean-value imputation	NLSVR	0.640	0.483	0.730	0.637
	Mode-value imputation	NLSVR	0.639	0.476	0.720	0.638
	iGMR-based imputation	RF	0.624	0.496	0.750	0.651
Dataset with time-series data but without squared terms and cross terms	Feature removal	EN	0.657	0.450	0.682	0.622
	Sample removal	PLS	0.733	0.423	0.642	0.559
	Zero-value imputation	EN	0.657	0.450	0.682	0.622
	Mean-value imputation	EN	0.659	0.448	0.679	0.620
	Mode-value imputation	EN	0.660	0.448	0.679	0.619
	iGMR-based imputation	EN	0.660	0.448	0.679	0.619
Dataset without time-series data but with squared terms and cross terms	Feature removal	RF	0.713	0.429	0.649	0.569
	Sample removal	GBDT	0.714	0.426	0.645	0.578
	Zero-value imputation	GBDT	0.724	0.412	0.623	0.558
	Mean-value imputation	GBDT	0.708	0.415	0.627	0.574
	Mode-value imputation	GBDT	0.729	0.403	0.609	0.553
	iGMR-based imputation	GBDT	0.729	0.409	0.619	0.553
Dataset with time-series data, squared terms, and cross terms	Feature removal	RF	0.699	0.440	0.665	0.583
	Sample removal	XGBoost	0.756	0.421	0.635	0.534
	Zero-value imputation	RF	0.707	0.437	0.660	0.575
	Mean-value imputation	RF	0.706	0.438	0.661	0.576
	Mode-value imputation	RF	0.702	0.441	0.666	0.580
	iGMR-based imputation	RF	0.708	0.433	0.654	0.574

Figure 3–6 reveal that adding time-series data or squared and cross terms caused the plots to cluster closer to the diagonal line, indicating improved prediction accuracy. As shown in Figs. 3 and 4, the inclusion of time-series data reduced both the mean absolute percentage error (MAPE) and the root mean square error (RMSE), demonstrating an improvement in prediction accuracy. Similarly, a comparison of Figs. 3 and 5 indicates that incorporating squared and cross terms also decreased MAPE and RMSE, while increasing the number of data points located near the diagonal line.

When both time-series data and squared and cross terms were included, both the MAPE and RMSE were small, with the RMSE reaching its minimum value, and the plots approached the diagonal line even in the low viscosity range. This indicates that accurate predictions can be achieved across the full product viscosity range with reduced variability. Therefore, the prediction model shown in Fig. 6 was adopted as the soft sensor. The soft sensor allows product viscosity to be predicted immediately after polymerization in the PVA production plant. This eliminates the time loss between polymerization completion and viscosity measurement.

### Design of process conditions

To develop a soft sensor for process condition design, only the initial condition variables determined prior to polymerization and other controllable variables should be used as explanatory variables. Uncontrollable factors, such as measurements obtained at the end of polymerization, cannot be used as explanatory variables. The variables selected using the Boruta algorithm included the ratio of newly supplied VAM and ester-type impurity (3) among the original variables. In addition, a large number of squared and cross-term features were associated with the ratio of newly supplied VAM, ester-type impurity (3), and conjugated-type impurity (6). These results indicate that feedstock and impurity information play an important role in determining product viscosity. Various regression analysis methods were used to construct soft sensors. Comparison of their prediction accuracies revealed that the RF method achieved the highest accuracy. Therefore, the soft sensor for process condition design was constructed using the RF regression method.

**Fig. 7:**
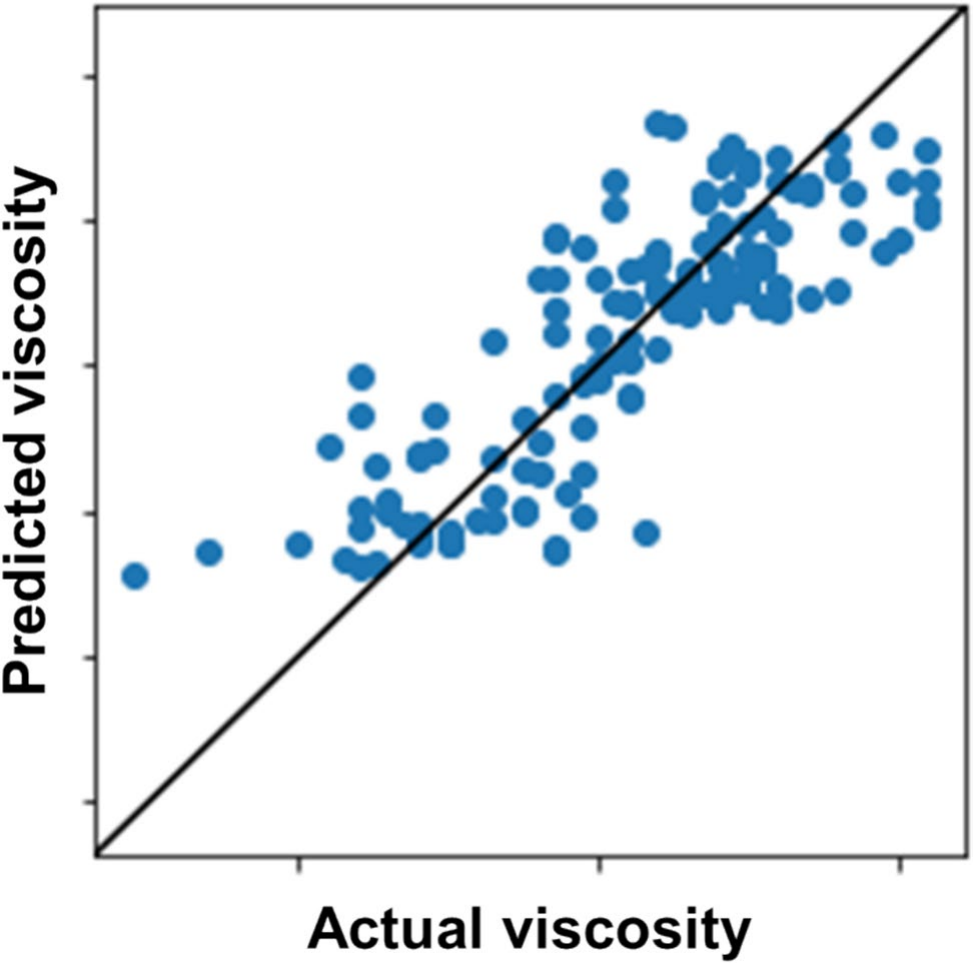
Plot of actual versus predicted values of product viscosity by a soft sensor for process condition design

Figure 7 shows a plot of actual versus estimated values for the soft sensor for process condition design, which was constructed using the RF regression method and evaluated using DCV. Compared with the soft sensor in Fig. 6, this soft sensor shows greater deviation from the diagonal line, indicating lower prediction accuracy. However, it has the advantage of being applicable to process condition design because it only uses initial condition variables and controllable variables.

**Fig. 8:**
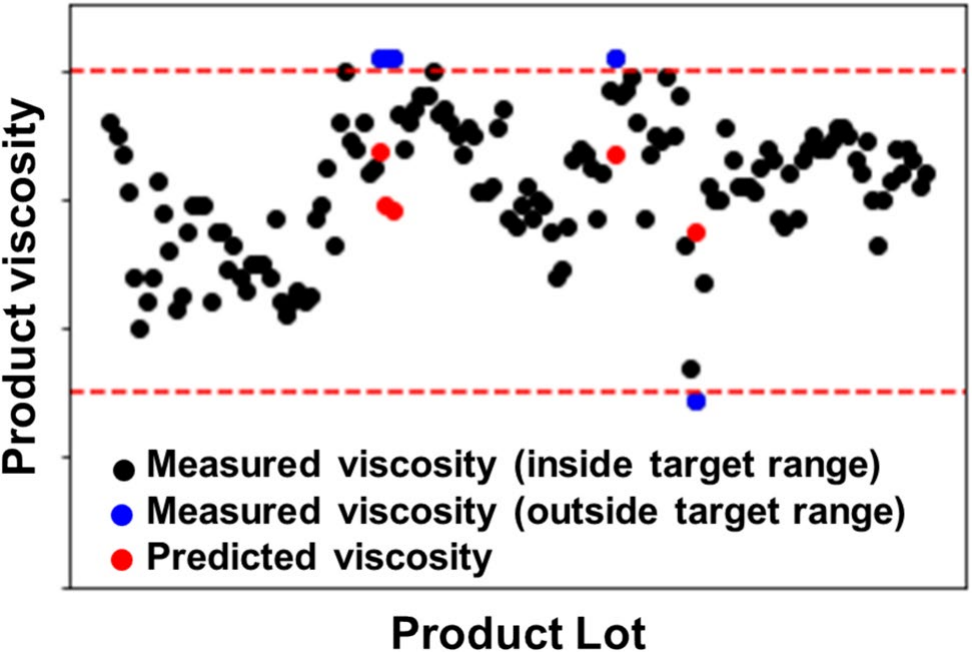
Measured and predicted product viscosity under optimal process conditions for all products with viscosities outside the target range. Black dots are measured viscosity values of products within the target range, blue dots are measured viscosity values of products outside the target range, red dots are predicted product viscosity values under optimal process conditions for products outside the target range, and red dashed lines are upper and lower limits of the target range. The horizontal axis is the product lot number and the vertical axis is the product viscosity

For products with viscosities outside the target range, we used a soft sensor for process condition design to determine optimal conditions that would bring the viscosity of the lot within the target range and predicted the resulting product viscosity under those conditions. In this analysis, the optimal process conditions were defined as those that produced a predicted viscosity closest to the median of the target range. The results are shown in Fig. 8. Using this soft sensor, we were able to design process conditions that brought the predicted viscosity within the target range for all such products.

## Conclusions

A soft sensor to predict product viscosity at the end of polymerization was developed with the aim of eliminating time loss between the end of polymerization and measurement of product viscosity. Additionally, process conditions were designed to ensure that product viscosity remained within the target range. To develop a soft sensor for accurate product viscosity prediction, we incorporated time-series data with squared and cross terms. For process condition design, case studies demonstrated that it was possible to determine optimal conditions to bring the product viscosity within the target range, even for products with viscosity outside the target range. As a result, this study achieved both real-time assessment of the process status through soft sensor-based viscosity prediction and active control of product viscosity through process condition design. In practical plant operations, product viscosity could be predicted using soft sensors after operating several batches, and if the predicted viscosity falls outside the target range, process conditions for the next batch could be designed and adjusted accordingly to maintain quality. Furthermore, by improving the prediction accuracy of the soft sensors for product viscosity and process condition design, the reliability of the predicted values and designed conditions can be further enhanced. Previous research [29] has shown that continuously updating a soft sensor model with new samples allows high prediction accuracy to be maintained. Therefore, implementing a model update scheme in this system can help prevent model degradation, enabling stable long-term operation.

## Data Availability

The authors do not have permission to share data.
